# Physical activity levels after low anterior resection for rectal cancer: one-year follow-up

**DOI:** 10.1186/s12889-021-12311-5

**Published:** 2021-12-13

**Authors:** Anne Asnong, André D’Hoore, Albert Wolthuis, Yves Van Molhem, Bart Van Geluwe, Annouschka Laenen, Nele Devoogdt, An De Groef, Tessa De Vrieze, Charlotte Van Calster, Inge Geraerts

**Affiliations:** 1 Department of Rehabilitation Sciences, Research Group Rehabilitation in Internal Disorders, KU Leuven – University of Leuven, Leuven, Belgium; 2 Department of Abdominal Surgery, University Hospitals Leuven and KU Leuven – University of Leuven, Leuven, Belgium; 3 Department of Abdominal Surgery, OLV Hospitals Aalst/Asse/Ninove, Aalst, Belgium; 4 Department of Abdominal Surgery, University Hospitals, Leuven, Belgium; 5 Interuniversity Centre for Biostatistics and Statistical Bioinformatics, KU Leuven and Hasselt University, Leuven, Belgium; 6 Center for lymphedema, University Hospitals Leuven, Leuven, Belgium; 7 Department of Rehabilitation Sciences, University of Antwerp, Antwerp, Belgium; 8 International Research Group Pain in Motion, Brussels, Belgium; 9 Department of Rehabilitation Sciences and Physiotherapy, MOVANT, University of Antwerp, Antwerp, Belgium

**Keywords:** Physical activity, Activities of daily living, Rectal neoplasms, Low anterior resection, Bowel symptoms, Predictive factors, Questionnaire

## Abstract

**Background:**

Overall survival rates after rectal cancer have increased. Therefore, functional outcomes rightly deserve more interest. The aims of this study were to assess progression in total, sports, occupational and household physical activity levels of rectal cancer survivors, from preoperatively to 12 months after surgery/stoma closure and to explore predictive factors.

**Methods:**

Multi-center prospective study with 125 patients who underwent low anterior resection for rectal cancer. The Flemish Physical Activity Computerized Questionnaire was completed concerning all physical activity levels at baseline (past preoperative year) and at 1, 4, 6 and 12 months after surgery/stoma closure. At these timepoints, questionnaires (LARS−/ COREFO-questionnaire) regarding bowel symptoms were also filled out. Results were analyzed using linear mixed models for repeated measures.

**Results:**

Total physical activity levels up to 12 months remained significantly lower than preoperative. Occupational and sports physical activity levels remained significantly lower until 6 and 4 months postoperative, respectively. Predictive factors for decreased physical activity levels at a specific timepoint were: younger age and no stoma (total physical activity, 1 month), low/mid rectal tumor, no stoma, non-employed status (total, 4 months), higher COREFO-scores (occupational, 4 months) and non-employed status (total, 12 months). At all timepoints, lower COREFO-scores were associated with higher total physical activity levels; male gender and lower educational levels with higher occupational levels; younger age, normal BMI, employed status and adjuvant therapy with higher sports levels; and female gender, lower educational level and unemployed status with higher household levels.

**Conclusions:**

One year after rectal cancer treatment, total physical activity levels were still not recovered. Rectal cancer patients, especially those at risk for decreased physical activity levels and with major bowel complaints, should be identified and guided to increase their activities.

**Trial registration:**

This trial has been registered at Netherlands Trial Register (NTR6383, 23/01/2017).

## Background

Worldwide, colorectal cancer (CRC) is the second most common cancer in women and the third most common cancer in men [1]. Almost 40% of these tumors find their origin in the rectum [1]. A low anterior resection (LAR) - more specifically a nerve-sparing, total mesorectal excision (TME) - remains the standard of surgical care in treating rectal cancer (RC). Due to improved treatment plans (including (neo) adjuvant radio- and/or chemotherapy), local recurrence rates are low [2] and overall survival have improved, albeit not without debilitating functional consequences. Between 60 and 90% of RC survivors experience the ‘Low Anterior Resection Syndrome’ (LARS) [3–5]. Recently, a consensus definition of LARS was formulated, which incorporates symptoms as well as consequences [6]. Following this definition, LARS is defined as the presence of at least one of the following symptoms: variable and unpredictable bowel function, altered stool consistency, increased stool frequency, repeated painful stools, emptying difficulties, urgency, incontinence or soiling. In order to classify these bowel symptoms as LARS, the presence of one or more of these symptoms should lead to an impact on predefined consequences, such as toilet dependence, dissatisfaction with bowels or mental and emotional wellbeing [6]. Therefore, greater emphasis on functional outcome improvement is necessary.

The impact of cancer treatment and its consequences on physical activity (PA) has been investigated in various studies [7–16]. PA concerns many different aspects: total, sports, occupational and household PA. PA levels are often investigated in the context of their capacity of reducing the risk of developing (C)RC. Results demonstrated that PA is associated with a reduced risk for colon cancer, but not for rectal cancer [9, 17]. Furthermore, most of these studies focused on only one of the aspects of PA and their interest was mainly targeting the effect of exercise interventions [8, 10, 11]. Higher PA levels were related to a better quality of life in (C)RC cancer survivors [7, 8]. However, to date, there are no studies describing the progression of the different aspects of PA during the first year after low anterior resection (LAR) in RC survivors. This is a major gap compared to other cancer populations [18–20].

Some patient-, treatment- and disease-related factors are associated with decreased PA levels after RC surgery. Lower levels of leisure-time PA were associated with older age, more years since surgery, lower educational level, lower income, and not having a partner [7]. Temporary stoma [21], neoadjuvant therapy [21], side-to-end coloanal anastomosis [22] and lower tumor height [23] were proven to influence the development of major LARS (symptoms like incontinence, frequency, clustering or urgency). Emmertsen et al. [21] stated that major LARS could have a negative effect on health in general and thus also on PA in RC survivors. Therefore, the aforementioned factors could be hypothesized to also negatively affect PA.

Thus, the aims of this study were to assess progression in total, sports, occupational and household PA levels of RC survivors, from preoperatively to 1, 4, 6 and 12 months after surgery/after stoma closure and to explore predictive factors for a decreased PA. We hypothesized that postoperative PA levels would not reach preoperative levels after treatment for rectal cancer and that the aforementioned predictive factors under investigation would have an effect on PA.

## Methods

This trial was approved by the local Ethical Committee of the University Hospitals Leuven (main Ethical Committee, reference s59761) and a positive advice from the Ethical Committees of the OLV Hospital Aalst and the General Hospital Groeninge Kortrijk was obtained. The trial was registered at Netherlands Trial Register (NTR6383, 23/01/2017).

This multi-center prospective study was conducted from January 2017–February 2021. All participants were recruited in Belgium at University Hospitals Leuven, OLV Hospital Aalst or General Hospital Groeninge Kortrijk. Patients who had a LAR (TME) for RC were eligible, but were excluded if they: (1) had another type of surgery for CRC, (2) were incontinent (faeces) before surgery, (3) had neurological diseases, (4) already had previous pelvic surgery, previous pelvic radiation or LAR for non-cancer reasons. Patients were included one month following surgery (LAR)/stoma closure (in case of a temporary stoma).

After consent, patients were asked to fill out the three following questionnaires: 1) the Flemish Physical Activity Computerized Questionnaire [24] (FPACQ) regarding PA and concerning bowel symptoms, 2) the Low Anterior Resection Syndrome Score [25] (LARS-questionnaire) and 3) the ColoRectal Functional Outcome questionnaire (COREFO-questionnaire) [26]. The FPACQ is a reliable and valid questionnaire to evaluate all PA levels (total, occupational, sports and household) [24]. Test-retest intraclass correlations of the FPACQ were good to excellent for both (un)employed/retired men and women. Furthermore, the FPACQ was found to be valid by comparing questionnaire-outcomes with results from the RT3 accelerometer [24]. All metabolic equivalent of task (MET) values used to calculate the PA variables were determined using the Ainsworth compendium of activities [27]. An overview of questionnaire characteristics can be found in Table 1 and calculation of each aspect of the FPACQ is explained with an example in Table 2. The LARS-questionnaire [25, 28] and the COREFO-questionnaire [26] were proven to be reliable for the assessment of bowel symptoms in this patient group. Furthermore, construct validity and criterion validity have been previously evaluated for the LARS-questionnaire [25, 28–30] and the COREFO-questionnaire as well [26, 30]. All patients prospectively completed the questionnaires within 2–7 days postoperatively (concerning preoperative year) and at 1, 4, 6 and 12 months after LAR/stoma closure. Patients were contacted by telephone if no response was received. A member of the research team checked for completeness upon receival of the questionnaires.

**Table 1:** Overview of questionnaire characteristics

	T**ool**	E**valuation of**	D**escription**	S**core**
Physical activity	FPACQ	physical activity and sedentary behavior during a usual week	one-week period domains: - patient-related data - data related to occupational activities: ○ occupational status ((un)employed) ○ working hours per week ○ job intensity ○ transport to the job - sport activities (3 most frequently performed sports): ○ frequency ○ duration - household activities ○ light ○ moderate ○ vigorous - transport during leisure time - sedentary activities (TV and sleep)	in MET-h/week: - total physical activity - occupational physical activity - sports physical activity - household physical activity
Bowel symptoms	LARS-questionnaire	Low Anterior Resection Syndrome symptoms	four-week period five questions with weighted score values	LARS-categories: - “no LARS” (0–20 points) - “minor LARS” (21–29 points) - “major LARS” (30–42 points)
	COREFO-questionnaire	functional outcome after colorectal surgery	two-week period 27 questions, score 0–4 for each question	between 0 and 100, with a higher score representing more symptoms

**Table 2:** Calculation of total, occupational, sports and household PA levels

D**efinition**	E**xample**	F**ormula**
occupational PA level	- A patient worked 38 h/week with 20% light, 70% moderate and 10% vigorous activities.	- occupational: (38 h/week × 20% × 2 MET) + (38 h/week × 70% × 3 MET) + (38 h/week × 10% × 4 MET) = 110 MET-hours/week
	- He drove 1.3 h/week for work by car.	- activity level for transport for work: 1.3 h/week × 1.5 MET = 2 MET-hours/week
sports PA level	This patient performed two sports.	(2 h/week × 5.5 MET) + (0.7 h/week × 3.5 MET) = 13 MET-hours/week
household PA level	He also performed 10 h/week light, 4 h/week moderate and 1.5 h/week vigorous household activities.	(10 h/week × 2.5 MET) + (4 h/week × 3.5 MET) + (1.5 h/week × 4.5 MET) = 46 MET-hours/week
total PA level		occupational + sports + household PA levels (*explained above*) + active transport in leisure time (1.0 h/week × 4 MET) + eating (8.8 h/week × 1.8 MET) + sleeping (49 h/week × 0.9 MET) + quiet leisure time (47.7 h/week × 1.5 MET) = 307 MET-hours/week

Using the definition for a MET as the ratio of work metabolic rate to a standard resting metabolic rate of 1.0 kcal/kg/h, one MET is considered as the resting metabolic rate during quiet sitting

### Predictive factors

Patient-, disease- and treatment-related factors were prospectively collected. Patient-related factors were age, gender, body mass index (BMI), partner status, educational level and employment status. These factors were collected as part of the FPACQ. Disease-related factors included tumor height (obtained from patient records) and bowel symptoms, inferred from the LARS-score and COREFO-questionnaire. Treatment-related factors included type of reconstruction (straight coloanal anastomosis, side-to-end coloanal anastomosis, J-pouch), (neo)-adjuvant therapy and stoma and were obtained from patient records at one month after LAR/stoma closure.

### Statistical analysis

Linear mixed models for repeated measures were used to evaluate the progression of continuous variables (total, occupational, sport and household activity levels) over time. Time was modelled as a categorical variable, whereas an unstructured covariance matrix is modelled to account for the correlation between repeated measurements. This approach has the advantage - compared to classical repeated measures ANOVA - that subjects with one or more missing measurements were still included in the analysis and that results were still valid when drop-out was missing at random [31]. Therefore, measurements without baseline values, could be included. Similar models were used to analyze the effect of various predictors (patient-, disease- and treatment-related factors) on the PA level and its evolution over time (baseline, 1, 4, 6 and 12 months postoperative). These models include time, predictor and the time by predictor interaction. A logistic regression model for repeated measures, with unstructured covariance matrix, was used to evaluate longitudinal binary measures such as job status or practicing sports. The analysis of the occupational PA level was restricted to preoperatively employed patients and the sports PA level to patients practicing some sports preoperatively. Patients, who retired during follow-up, were only considered at the time points they were still employed.

Tukey–Kramer or Holm adjustment was used for multiple post hoc comparisons. Analyses have been performed using SAS software (version 9.4, SAS System for Windows), a *P* value < 0.05 was considered statistically significant.

## Results

One hundred twenty-five patients were included. Concerning the PA level one year before surgery, 120 patients filled out the FPACQ. At 1, 4, 6 and 12 months, 121 (96.8%), 113 (90.4%), 105 (84.0%) and 101 (80.8%) patients filled out the questionnaire, respectively. Baseline characteristics can be found in Table 3.

**Table 3:** Baseline characteristics (*n* = 125) and bowel complaints

Variable	Value	
	mean (SD)/ median (IQR)	n (%)
Age, years, mean (SD)	58.49 (11.07)	
≤ 49 years		23 (18.40)
50–69 years		82 (65.60)
≥ 70 years		20 (16.00)
Gender		
Male		83 (66.40)
Female		42 (33.60)
BMI, kg/m^2^, median (IQR)	24.58 (22.77–27.48)	
< 25.0		69 (55.20)
25.1–30.0		39 (31.20)
> 30.0		17 (13.60)
Partner		
Yes		109 (87.20)
No		16 (12.80)
Educational level		
Semi−/unskilled		72 (57.60)
Highly skilled		53 (42.40)
Employment status		
Retired		54 (43.20)
Employed		61 (48.80)
Unemployed		10 (8.00)
Tumor height^a^		
Low (0–5 cm)		67 (53.60)
Mid (6–10 cm)		39 (31.20)
High (11–15 cm)		19 (15.20)
LARS-score (*n* = 124)		
No		81 (66.39)
Minor		20 (16.39)
Major		21 (17.21)
COREFO-score, median (IQR) (*n* = 122)	5.77 (1.92–12.50)	
Type of reconstruction		
Straight coloanal anastomosis		73 (58.40)
Side-to-end coloanal anastomosis		33 (26.40)
Colon pouch-anal anastomosis/J-pouch		19 (15.20)
Neoadjuvant therapy		
No		41 (32.80)
Chemo- and/or radiotherapy		84 (67.20)
Adjuvant therapy		
No		67 (53.60)
Chemotherapy		56 (44.80)
Chemoradiotherapy		2 (1.60)
Stoma (duration of 178 days (± 108))		
Yes		107 (85.60)
No		18 (14.40)

^a^ from the anal verge

### Progression of PA

**Fig. 1:**
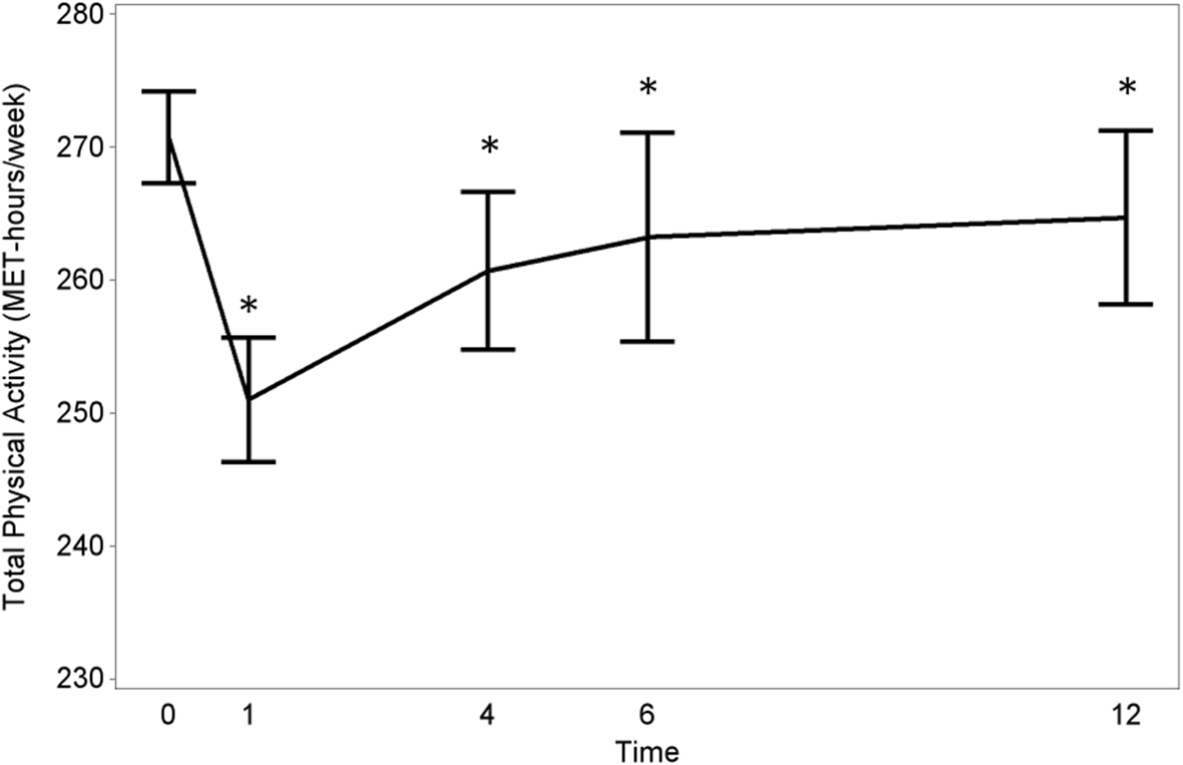
Progression of the total PA (MET-hours/week) levels of RC patients from the preoperative stage to 12 months after surgery/stoma

An overview of the results of the progression of PA is provided in Table 4. The total PA levels decreased by 8.5% (from 274.21 MET-h/week preoperatively to 251.00 MET-h/week) at one month after LAR (or stoma closure). At 4, 6 and 12 months after LAR/stoma closure, total PA levels were respectively 4.9, 4.0 and 3.5% lower than baseline (Fig. 1).

**Table 4:** Progression of PA levels over time

PA	P**reoperative** (***n*** = 120)	1 **month** (***n*** = 121)	4 **months** (***n*** = 113)	6 **months** (***n*** = 105)	12 **months** (***n*** = 101)
Total PA:					
Mean estimate, MET-h/week	274.21	251.00	260.70	263.25	264.74
95% CI	267.29–281.12	246.32–255.67	254.77–266.63	255.37–271.13	258.21–271.27
P		<.001*	<.001*	0.048*	0.046*
Occupational PA:					
Mean estimate, MET-h/week	106.22	79.35	84.57	89.53	95.23
95% CI	97.17–115.27	59.01–99.68	72.58–96.56	78.51–100.55	84.77–105.69
P		0.030*	0.001*	0.017*	0.058
Sports PA:					
Mean estimate, MET-h/week	17.18	3.98	8.42	11.71	11.58
95% CI	13.42–21.91	2.48–6.13	5.57–12.49	7.95–17.05	7.64–17.32
P		<.001*	0.002*	0.259	0.277
Household PA:					
Mean estimate, MET-h/week	34.45	30.63	32.40	29.35	33.14
95% CI	30.14–38.77	25.86–35.40	28.17–36.62	25.61–33.09	28.60–37.69
P		0.476	0.853	0.075	0.972

P value of pairwise differences between preoperative and the particular point in time

* *P* value < 0.05

**Fig. 2:**
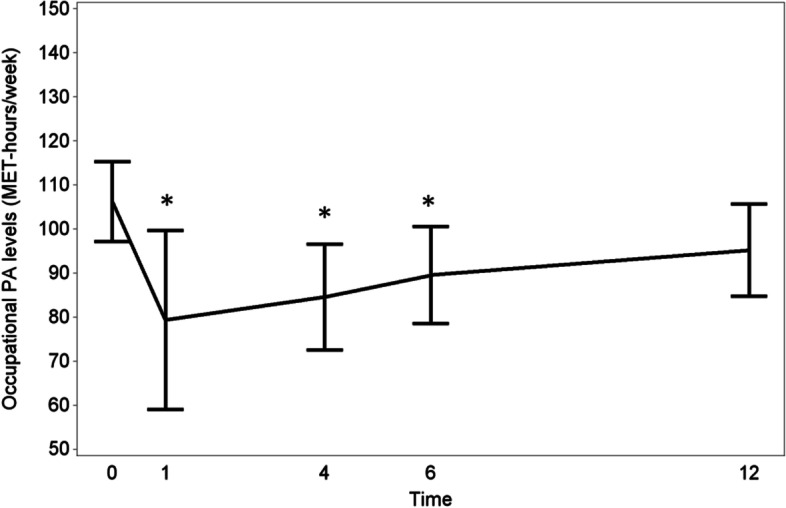
Progression of the occupational PA (MET-hours/week) levels of RC patients from the preoperative stage to 12 months after surgery/stoma

A similar trend was found for occupational and sports PA levels. Patients who were employed before LAR (*n* = 60) spent 25% less MET-hours on occupational activities at 1 month. At 4, 6 and 12 months, a decrease of respectively 20, 16 and 10% in occupational PA levels was recorded (Fig. 2). Of the non-retired patients (*n* = 71), 60 (85%) were employed before surgery. At 1, 4, 6 and 12 months, 31, 42, 48 and 52% had resumed their professional activities.

**Fig. 3:**
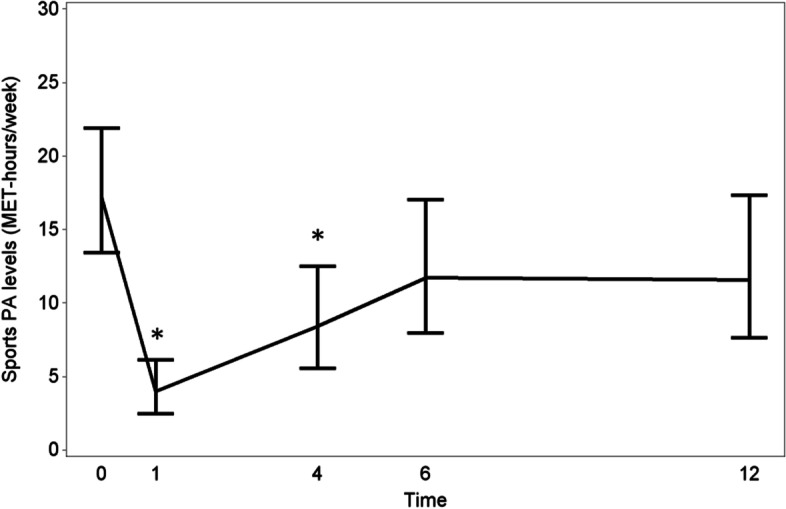
Progression of the sports PA (MET-hours/week) levels of RC patients from the preoperative stage to 12 months after surgery/stoma

In patients that were performing sports before surgery (*n* = 70), the sports PA level reduced with 77% at one month postoperatively (Fig. 3). Decreases of 51, 32 and 33% were noted for 4, 6 and 12 months postoperative, respectively. Of the preoperatively sport-active patients (n = 70), 59, 77, 82 and 80% were practicing sport activities at 1, 4, 6 and 12 months. From patients that were not sport-active before surgery, 31% did practice some sport activity at 12 months.

Postoperative household PA levels remained relatively stable compared to preoperative levels and no significant results were found.

### Predictive factors for progression of PA levels

**Fig. 4:**
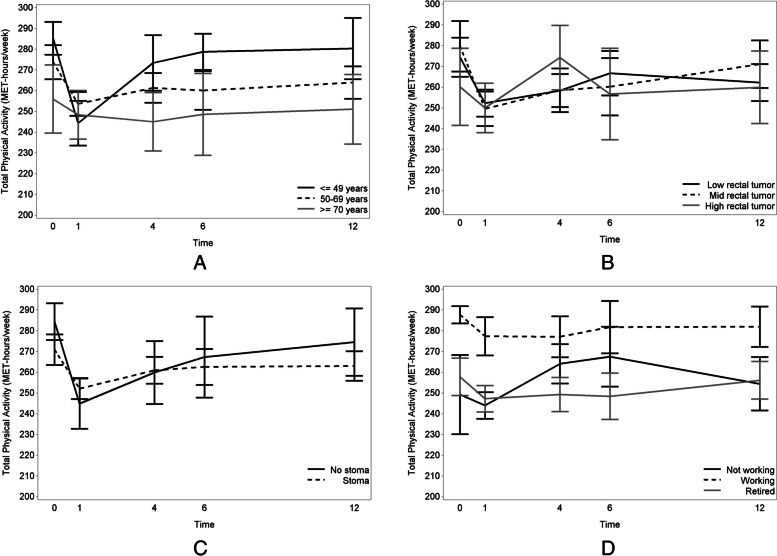
Predictive factors for total PA levels from the preoperative stage to 12 months after surgery/stoma closure

Overall, the total PA level was not significantly influenced by age, tumor height or stoma at the different timepoints. However, younger patients had a greater decrease in total PA level than older patients (*p* = 0.007 and *p* = 0.002) at 1 month after surgery/stoma closure (Fig. 4A). Additionally, patients with a low (*p* = 0.013) or mid (*p* = 0.005) rectal tumor, had a larger decrease in total PA level at 4 months than patients with a high rectal tumor (Fig. 4B) and patients with a stoma had a stronger decrease in total PA level at 1 (*p* = 0.004) and 4 months (*p* = 0.019) (Fig. 4C). Furthermore, total PA levels were higher for working patients compared to retired patients at all points in time (*p* < 0.001 at 1 month to *p* = 0.014 at 12 months). Total PA levels for working patients compared to non-working patients (non-employed and sick/disabled patients) were only significantly higher at baseline (p = 0.004), 4 (p < 0.001) and 12 (*p* = 0.031) months postoperatively/after stoma closure (Fig. 4D). Consequently, the effect of bowel symptoms (COREFO-scores) on total PA level was not different at the various time points, indicating a general effect. In particular, higher COREFO-scores were shown to be associated with lower total PA levels (*p* = 0.002). For every increase of one point on the COREFO-score, the total PA decreased with 0.24 MET-h/week.

**Fig. 5:**
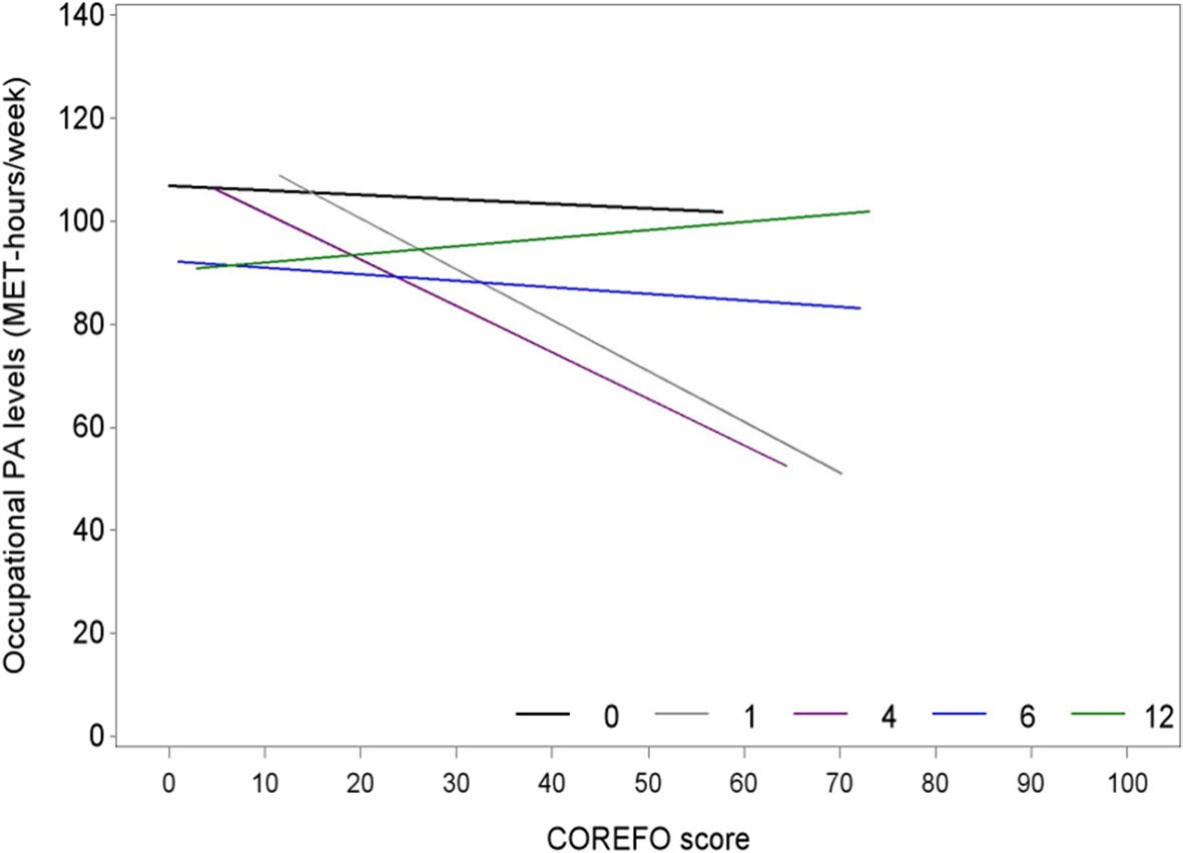
Predictive factor for the progression of occupational PA levels from the preoperative stage to 12 months after surgery/stoma closure

The occupational PA level of male patients (*p* = 0.001) and patients with a lower educational level (*p* = 0.037) is significantly higher than for females or patients with a higher educational level at all timepoints. Furthermore, the amount of bowel symptoms had a significant effect on the absolute level of occupational PA only at 4 months. For every increase of one point on the COREFO-score at 4 months (*p* = 0.040) a patient was 0.9 MET-h/week less physically active (Fig. 5).

Regarding household PA levels, females (*p* = 0.003), lower educated patients (*p* < 0.001) and unemployed patients (*p* = 0.035) had significantly higher household PA levels than males, higher educated patients and employed patients, at every time point (1, 4, 6 and 12 months after LAR/stoma closure).

Concerning sports PA levels, younger patients (age < 50 years; p = 0.040), patients with a normal BMI (≤25 kg/m^2^; *p* = 0.001), employed patients (*p* = 0.013) and patients who underwent adjuvant therapy (*p* = 0.026) showed higher activity levels than older (age 50–70 years), overweight (25–30 kg/m^2^), non-employed patients or patients without adjuvant therapy, at every timepoint (1, 4, 6 and 12 months after LAR/stoma closure).

All patient-, disease-, and treatment-related factors (cfr. Methods section) were investigated for all aspects of PA. Due to the multitude of data, only significant predictors were discussed.

## Discussion

In this study, a decrease of 8.5% of the total PA level was found one month after LAR/stoma closure for RC. This decrease remained significantly lower up to one year after surgery/stoma closure, emphasizing the impact of the RC treatment on patients’ PA. In general, literature in the context of RC and its association with PA after surgery is very scarce and most of the literature on functional consequences views RC and CRC survivors as one group. The same applies for rehabilitation programs in RC and CRC survivors. A recent systematic review of Balhareth et al. [32] showed that PA has been determined to positively impact the quality of life after CRC, but additionally showed also a lack of consensus on guidelines and conclusive evidence on the content and form of said rehabilitation programs.

To the best of our knowledge, no previous studies investigated the evolution of PA during the first year after LAR/stoma closure in RC patients. Nonetheless, PA was investigated in relation to health-related quality of life (HRQOL) in long-term RC survivors (≥ 5 years) [7]. Other studies focused on the effects of (pre) rehabilitation programs for RC patients, in which possible positive effects on physical fitness [10, 11] and tumor regression [10] were highlighted. Additionally, most studies only focused on one aspect of PA. The decrease in total, as well as occupational and sports PA levels in our study can be explained by the fact that approximately half of the patients underwent adjuvant therapy after surgery, which has previously been found to be associated with declined exercise levels in CRC patients [12, 13]. For occupational PA levels, the decrease up to 6 months is in line with previous research stating that work ability was decreased at 3 and 6 months after curative treatment for RC and return to work delayed [33, 34]. For household PA levels, no significant decreases were found in the postoperative progression of RC survivors.

Regarding predictive factors in the present study, the total PA level of younger patients (age < 50 years), patients with a low/mid rectal tumor and patients with a stoma did not significantly differ from respectively older patients, patients with a high rectal tumor or patients without a stoma at the different timepoints. However, being younger was a predictive factor for a decreased total PA at one month. No previous research was found on predictive factors for total PA after RC. Secondly, patients without a stoma had a significantly greater decrease at one and four months after LAR, compared to baseline. Hence, it should be mentioned that patients without a stoma were included one month after their major surgery (LAR), while patients with a stoma were included at one month after stoma closure, i.e. after a longer timeframe (on average 178 days) since their LAR. Inclusion of patients at these specific timepoints was chosen because of the common interest in the impact of LAR on bowel function. Firstly, previous research has shown that LARS usually manifests in a period of one month after transit recovery [35]. Secondly, the interval from surgery to stoma closure was not associated with LARS [36]. Consequently, including patients only based on time after LAR would have prevented conclusions regarding the influence of bowel dysfunction on PA. Previous work has shown that stoma-related challenges (pouch leakages, skin irritation, risk of hernia, self-consciousness…) have a negative impact on patients’ engagement in PA [14, 15]. The lower total PA levels from 6 months onwards in patients who had a stoma could therefore be partially explained by persisting diminished PA due to the previous challenges, even after stoma closure. The significant decrease in total PA early after LAR patients without a stoma, might be explained by the influence of adjuvant therapy [12]. Furthermore, an interaction effect was found between tumor height and time, although this might be due to an inexplicable spike in the total PA at 4 months, in survivors with a high tumor. No literature was found regarding the predictive value of tumor height on PA levels. Concerning employment status, a significant interaction was found as well; probably due to an increase in total PA at 4 and 6 months in RC survivors who were not working due to unemployment or sickness/disability. This increase might be explained by a within-person change in employment status in patients who worked preoperatively. Bowel complaints could have forced employees to stay at home, as was stated in previous long-term research [34]. While they were not working at follow-up, their previous activity levels might have remained consistent. Lastly, higher COREFO-scores were correlated with lower total PA overall. Consequently, higher COREFO-scores were also predictive for lower occupational PA levels at 4 months after LAR/stoma closure. This was confirmed in previous research regarding the link between major LARS and the (in) ability to work [21] as well as long-term findings [34]. Furthermore, females and higher educated patients had generally lower levels of occupational PA overall. Lynch et al. [13] formerly confirmed this for PA in general after CRC.

For household PA levels, a higher educational level was also associated with lower PA, analogous to the results mentioned for occupational PA. For gender, however, being male was predictive for lower household PA levels, which might be linked to stereotypical gender roles concerning household work.

For sports PA levels, older age, being overweight or being unemployed/sick/disabled were predictive for generally lower PA levels after RC, which was in line with previous research stating that younger RC survivors reported more leisure-time PA [7] and increasing weight was associated with physical inactivity in cancer survivors in general [16]. No previous research was found on the predictive value of employment status.

The present study has many strengths. This is the first study to investigate the progression of all aspects of PA (total, occupational, sports and household). Patients were assessed before surgery regarding PA over the past year and at fixed time intervals after surgery/stoma closure up to 12 months. The return rate of the FPACQ was very high (81–96% at the various timepoints). Furthermore, this study is unique in finding a predictor of return to PA in bowel symptoms after LAR for rectal cancer. In particular, the majority of patients filled in the LARS- (80–99%) and COREFO- (82–98%) questionnaire at the various timepoints. All patients were operated in high-volume hospitals by very experienced surgeons, which enables generalization of the results. A limitation of the present study was that the FPACQ was proven to be reliable and valid in healthy adults but not specifically in RC patients. Furthermore, notwithstanding the validity and reliability, a weakness lies in using the FPACQ for the evaluation of PA- a questionnaire - which remains a subjective measurement method. Lastly, the results in this study should be interpreted with care given the multitude of models and *P* values.

## Conclusion

This is the first study to investigate the progression of PA as a whole (total, occupational, sports and household PA levels) after RC treatment, to find predictive factors for a decrease in PA after LAR for RC and to assess the impact of bowel symptoms on PA. All aspects of PA, except household PA, decreased significantly until 4 months after surgery/stoma closure. Occupational PA and total PA decreased even until respectively 6 and 12 months. Predictive factors for decreased PA levels at a specific timepoint were: younger age and no stoma (total PA, 1 month), low/mid rectal tumor, no stoma, non-employed status (total PA, 4 months), higher COREFO-scores (occupational PA, 4 months) and non-employed status (total PA, 12 months). Furthermore, at all timepoints, lower COREFO-scores were associated with higher total PA levels; male gender and lower educational levels with higher occupational levels; younger age, normal BMI, employed status and adjuvant therapy with higher sports levels; and female gender, lower educational level and unemployed status with higher household levels. RC patients, especially those at risk for decreased PA levels and those with major bowel complaints, should be well guided to minimalize the decrease in PA levels and regain preoperative levels as soon as possible.

## Data Availability

The data that support the findings of this study are available from the corresponding author (anne.asnong@kuleuven.be) upon reasonable request.

## Abbreviations

BMI: Body mass index
CRC: Colorectal cancer
COREFO: ColoRectal Functional Outcome questionnaire
FPACQ: Flemish Physical Activity Computerized Questionnaire
LAR: Low anterior resection
LARS: Low Anterior Resection Syndrome
LARS-questionnaire: Low Anterior Resection Syndrome Score
MET: Metabolic equivalent of task
PA: Physical activity
RC: Rectal cancer
TME: Total mesorectal excision
